# Association of the retinal vasculature, intrathecal immunity, and disability in multiple sclerosis

**DOI:** 10.3389/fimmu.2022.997043

**Published:** 2022-11-11

**Authors:** Christina Noll, Michael Hiltensperger, Lilian Aly, Rebecca Wicklein, Ali Maisam Afzali, Christian Mardin, Christiane Gasperi, Achim Berthele, Bernhard Hemmer, Thomas Korn, Benjamin Knier

**Affiliations:** ^1^ Department of Neurology, Klinikum rechts der Isar, TUM School of Medicine, Technical University of Munich, Munich, Germany; ^2^ Institute for Experimental Neuroimmunology, TUM School of Medicine, Technical University of Munich, Munich, Germany; ^3^ Munich Cluster of Systems Neurology (SyNergy), Munich, Germany; ^4^ Department of Ophthalmology, University Hospital of Erlangen-Nuremberg, Erlangen, Germany

**Keywords:** Optical coherence tomography, optical coherence tomography angiography, multiple sclerosis, cerebrospinal fluid, disability

## Abstract

**Background:**

Optical coherence tomography angiography (OCT-A) is a novel technique allowing non-invasive assessment of the retinal vasculature. During relapsing remitting multiple sclerosis (RRMS), retinal vessel loss occurs in eyes suffering from acute optic neuritis and recent data suggest that retinal vessel loss might also be evident in non-affected eyes. We investigated whether alterations of the retinal vasculature are linked to the intrathecal immunity and whether they allow prognostication of the future disease course.

**Material and methods:**

This study includes two different patient cohorts recruited at a tertiary German academic multiple sclerosis center between 2018 and 2020 and a cohort of 40 healthy controls. A total of 90 patients with RRMS undergoing lumbar puncture and OCT-A analysis were enrolled into a cross-sectional cohort study to search for associations between the retinal vasculature and the intrathecal immune compartment. We recruited another 86 RRMS patients into a prospective observational cohort study who underwent clinical examination, OCT-A and cerebral magnetic resonance imaging at baseline and during annual follow-up visits to clarify whether alterations of the retinal vessels are linked to RRMS disease activity. Eyes with a history of optic neuritis were excluded from the analysis.

**Results:**

Rarefication of the superficial vascular complex occured during RRMS and was linked to higher frequencies of activated B cells and higher levels of the pro-inflammatory cytokines interferon-γ, tumor necrosis factor α and interleukin-17 in the cerebrospinal fluid. During a median follow-up of 23 (interquartile range 14 - 25) months, vessel loss within the superficial (hazard ratio [HR] 1.6 for a 1%-point decrease in vessel density, p=0.01) and deep vascular complex (HR 1.6 for a 1%-point decrease, p=0.05) was associated with future disability worsening.

**Discussion:**

Optic neuritis independent rarefication of the retinal vasculature might be linked to neuroinflammatory processes during RRMS and might predict a worse disease course. Thus, OCT-A might be a novel biomarker to monitor disease activity and predict future disability.

## Introduction

Alterations of the optic nerve and the retina are frequent findings in patients with multiple sclerosis (1). Optical coherence tomography (OCT) allows high-resolution visualization of the retinal architecture and is increasingly applied to study retinal pathology during multiple sclerosis. Here, atrophy of the inner retinal layers, in particular of the combined ganglion cell and inner plexiform layer (GCIP), has been linked to neurodegenerative processes of the central nervous system and is associated with a worse disease prognosis (2–4).

OCT angiography (OCT-A), a functional extension of the conventional OCT technique, facilitates rapid, non-invasive and high-resolution imaging of retinal blood vessel structures. It acquires consecutive scans at one location of the retina, and after removal of stationary tissue signals, the remaining signal reflects the corpuscular blood constituents in both venous and arterial blood vessels (5). There is growing evidence that patients suffering from autoimmune diseases of the central nervous system also reveal alterations of the retinal vasculature. We could recently show that an acute optic neuritis (ON) leads to loss of retinal vessels within the superficial vascular complex (SVC) during the first 90 days after ON (6). Superficial vessel loss might also occur in eyes without a history of ON and might be linked to a worse visual function and disability (7, 8). Underlying mechanisms, however, are not fully understood and mechanistic models of retinal pathology in CNS autoimmune inflammation are in their infancy (9, 10).

In the current study, we firstly investigated whether alterations of the retinal vasculature are linked to changes in the cellular and humoral immune compartment in the cerebrospinal fluid (CSF) of patients with early relapsing remitting multiple sclerosis (RRMS). Secondly, we investigated potential associations between retinal vessel loss and future disease activity and disability.

## Material and methods

### Study design

The current study includes two different patient cohorts consisting of one cross-sectional (CSF cohort) and one prospective observational cohort (clinical cohort) of patients with RRMS according to the McDonald criteria 2017 (11) between 17 and 65 years of age and one cohort of healthy controls (HC cohort). All cohorts were recruited at the Department of Neurology, Klinikum rechts der Isar, TUM School of Medicine of the Technical University of Munich between 2018 and 2020.

We recruited patients receiving lumbar puncture for diagnostic work-up of suspected MS into a cross-sectional cohort study. Patients underwent anamnesis, clinical examination including assessment of the expanded disability status scale (EDSS), lumbar puncture with CSF analysis, retinal OCT, and OCT-A within two days after study inclusion. The main purpose of this study cohort was to investigate potential associations between OCT-A measures and alterations of intrathecal immunity.

We enrolled further patients with clinically isolated syndrome (CIS) and RRMS into a prospective observational cohort study. At baseline, we took a detailed medical history and patients received a clinical examination, OCT, OCT-A, and cerebral MRI. Baseline visits took place at least 30 days after the last clinical relapse. Follow-up visits were scheduled depending on the clinical presentation. All patients received an EDSS assessment and a MRI scan at least once per year. Disease modifying therapies (DMT) were categorized as DMT class 1 (dimethyl fumarate, glatiramer acetate, interferon beta-1a/b, teriflunomide), DMT class 2 (cladribine, fingolimod) and DMT class 3 (alemtuzumab, natalizumab, ocrelizumab), and “no DMT” if patients did not receive any DMT. The primary outcome parameter was confirmed disability worsening as measured by EDSS. EDSS worsening was defined as an increase in the EDSS score ≥ 1.5 in patients with an EDSS = 0, ≥ 1.0 points in patients with an EDSS between 1.0 and 5.0, and ≥ 0.5 points in patients with an EDSS ≥ 5.5, each confirmed by a visit at least 3 months later. EDSS score assessment occurred at least 30 days distant to any occurred relapses. Secondary outcome parameters were annualized relapse rate (ARR) and MRI disease activity defined as an increase in T2 lesion load. The main purpose of this cohort was to search for OCT and OCT-A predictors of the future disease course.

Moreover, we selected healthy individuals from an already existing healthy control (HC) cohort in our department. Here, individuals were age- and sex-matched to both the CSF and clinical cohort. The HC cohort consists of mainly employees and students from our department. All HC were screened for defined comorbidities by thorough anamnesis but without a neurological or ophthalmological examination. The main purpose of this cohort was to provide healthy and physiological OCT-A and OCT reference measures.

In all three cohorts, exclusion criteria were substantial eye disease that may affect the integrity of the retinal architecture or vasculature, refractory errors > 6 diopters, and poor OCT-A quality in both eyes. Individual eyes with a history of ON, suspected subclinical ON or poor OCT-A quality were removed from the analysis.

### Standard protocol approvals, registrations, and patient consents

We followed STROBE guidelines for reporting observational studies. The study was approved by the ethics committee of the Technical University of Munich, School of Medicine (116/16 S, 9/15 S), and adhered to the Declaration of Helsinki. All participants gave written informed consent.

### Data availability

Data are available upon reasonable request from the corresponding author. We will share raw imaging OCT-A data in an anonymized way upon request by any qualified investigator. The data are not publicly available due to privacy or ethical restrictions.

### OCT and OCT-A analysis

Conventional OCT images were acquired as described elsewhere (3) and included examination of the peripapillary retinal nerve fiber layer (pRNFL) and the macula (30° x 25° macular scan). We checked all scans for sufficient quality according to the OSCAR-IB criteria (12). Retinal segmentation was performed automatically by an inbuilt software algorithm (Eye Explorer, v2.5.4.) and was manually corrected if necessary.

OCT-A examinations were acquired on both eyes of each patient under low-lighting conditions using a spectral-domain OCT with angiography module (Heidelberg Engineering Spectralis OCT2) by two experienced technicians as previously described (6). En face images and decorrelation signals were recorded within a 2.9 × 2.9 mm area focusing on the fovea centralis with active eye tracking algorithm. Segmentation of the macular area was done automatically by the in-built software (v2.5.4) into the SVC and the deep vascular complex (DVC). For analysis of retinal vessel density measures, we applied the Erlangen Angio Tool (13) as described in detail elsewhere (6). Here, SVC and DVC vessel densities were assessed within a circle around the fovea between 0.8 mm and 2.9 mm eccentricity (area 6.1 mm²). Quantification of the foveal avascular zone (FAZ) was calculated using a MatLab (MathWorks, vR2019b) algorithm as described elsewhere (6). To ensure sufficient OCT-A image quality, we performed a thorough and standardized assessment of OCT-A image quality. We excluded OCT-recordings with 1) obvious problems (like retinal pathology, defocus, tilting, opacities), 2) a poor signal strength of Q < 30, 3) decentration of the imaging focus, 4) incorrect segmentation of the vascular complexes, which was assessed manually within the respective B scans, 5) major motion artifacts defined as a motion artifact score (14) > 2 (15) and 6) OCT-A recordings with major projection artifacts. Eyes with former clinical and unilateral subclinical ON were excluded from the analyses. A history of unilateral subclinical ON was defined as an inter-eye difference of both the pRNFL and the GCIP of more than 5 and 4 µm, respectively (16).

### Assessment of the visual function

Monocular visual acuity was measured at high (100%) and low (2.5%) contrast using Early Treatment Diabetic Retinopathy Study (ETDRS) charts. Charts were placed in a retro-illuminated cabinet (Precision Vision, USA) with 80 candela per square meter in 2 meters distance. Visual acuity was tested with best refractive correction according to the manufacturer’s specifications. Visual acuity was calculated from the smallest correctly read line as the decimal value of the Snellen faction.

### CSF analysis

We performed CSF and blood sampling prior to initiation of any DMT. Samples were immediately processed after collection. Albumin and immunoglobulin (Ig) G, IgM, and IgA were measured in cell free CSF and serum using a BNProSpec (Siemens) analyzer after centrifugation. CSF supernatants were frozen at -80°C and stored in the biobank of the Department of Neurology for further analysis. For cellular analyses, fresh CSF cells were washed with 2% fetal calf serum/phosphate-buffered saline and directly processed on ice. Surface staining was performed using anti-CD3-Pacific blue, -CD4-PE, -CD8-FITC, -CD14-APC-eF780, -CD19-ECD, -CD20- APC-eF780, -CD27-APC, -CD38-APC-A700, -CD45-BV510, -CD56-PE-Cy7, -CD138-BV605 and anti-HLADR-PerCP antibodies (Beckman Coulter, ThermoFisher, Biolegend) for 30 minutes at 4°C. Samples were analyzed directly after the staining procedure using a CytoFLEX flow cytometer (Beckman Coulter) and FlowJo (v10.8.1) software. Gating strategies and analyzed cell populations are shown in the **Supplementary Figure 1**.

For analysis of humoral mediators, we thawed frozen cell free CSF on ice and applied two different flow cytometry-based multiplex assays (Biolegend, LEGENDplex multiplex assays). All samples of all patients were processed simultaneously and as suggested by the manufacturer. Measurements were run in duplicates. The T helper cytokine panel allowed quantification of interleukin (IL-) 2, IL-4, IL-5, IL-6, IL-9, IL-10, IL-13, IL-17A, IL-17F, IL-21, IL-22, interferon-γ (IFN-γ), and tumor necrosis factor α (TNF-α). The human B cell activator panel allowed assessment of levels of the B cell activator factor (BAFF), a proliferation inducing ligand (APRIL), and CD40 ligand (CD40L) in CSF. Minimal detectable concentrations as suggested by the manufacturer are provided within the **Supplementary Table 2**.

### Statistical analysis

We performed statistical analyses with GraphPad Prism (v9.3.1). To account for inter-eye correlations, mean values of both eyes were used as one data point when both eyes were available. If one eye was excluded, values of the remaining eye were used. Quantitative differences between two groups were calculated using an unpaired t test if values were normally distributed and a non-parametric Mann–Whitney U test if not. Multiple linear regression models were used to search for associations between OCT-A values on clinical disease and CSF patterns. We corrected all models for the covariates age, sex, disease duration, and EDSS if not otherwise stated and provided the respective estimates (ß-value) as regression parameters. To analyze the association of baseline OCT-A parameters and primary and secondary outcome measures, we applied Cox proportional hazards regression models. We adjusted Cox proportional hazards regression models for age, sex, and DMT during the follow-up period if not otherwise stated and provide hazard ratio measures (HR). To identify OCT-A values that differentiate best between different outcome measures, we performed receiver operating characteristics (ROC) and provided the respective area under the ROC curve (AUC) results. Values are provided as mean ± standard deviation (SD) if normally distributed or otherwise as median (25%-75% interquartile range; IQR). The statistical significance threshold was p<0.05.

## Results

### Study cohorts and baseline characteristics

The CSF cohort consisted of 90 patients with a median age of 35 (29 – 46) years and a disease duration of 0 (0 – 17) months (**Table 1**). We excluded 35 eyes from further analysis due to acute ON and another 7 eyes due to suspected unilateral subclinical ON. Another 18 eyes were excluded due to poor OCT-A quality, resulting in 120 eyes of 90 patients that were included into the study (**Supplementary Figure 2**). Here, OCT-A data was available in 87 patients and OCT measures in all of the 90 enrolled patients. All patients underwent diagnostic workup for first diagnosis of suspected multiple sclerosis and most patients (86%) suffered from an acute relapse at study inclusion. The different type of relapses and affected neurological systems are described in the **Supplementary Table 1**. Of those patients, who suffered from acute relapse and received steroid therapy (**Table 1**), spinal tap and CSF analysis was performed before initiation of steroid treatment in 58/67 patients (87%). No patient received any DMT for treatment of multiple sclerosis at the time of CSF analysis. As shown in **Table 1**, patients from the CSF cohort revealed an inflammatory CSF phenotype with intrathecal IgG synthesis, presence of CSF-specific oligoclonal bands in the majority of patients, and increased B cell frequencies (17).

**Table 1:** Clinical and CSF characteristics of the CSF cohort.

CSF cohort (n=90)	
Demographics	
Age, years	35 (29 – 46)
Sex female, No. (%)	62 (69)
Disease duration, months	1 (0 – 17)
EDSS	2.0 (1.0 – 3.0)
Relapse, No. (%)	77 (86)
Steroid therapy, No. (%)	67 (74)
Diagnosis, No. (%) - Clinically isolated syndrome - Relapsing remitting multiple sclerosis	11 (12) 79 (88)
Disease modifying therapy, No. (%)	0 (0)
**CSF nephelometry/electrophoresis**	
Albumin, CSF-serum index x 10^-3^	5.3 (4.2 – 6.5)
IgG, CSF-serum index	0.73 (0.54 – 1.03)
IgA, CSF-serum index	0.28 (0.25 – 0.32)
IgM, CSF-serum index	0.07 (0.04 – 0.17)
Presence of oligoclonal bands, No. (%)	78 (87)
**CSF cytometry**	
Cell count, per µl	4 (2 – 11)
CD4^+^ T cells, %^#^	68.7 (65.8 – 73.8)
CD8^+^ T cells, %^#^	17.3 (14.1 – 20.5)
CD19^+^ B cells, %^#^	2.3 (1.6 – 4.0)
CD19^+^CD138^+^ plasma cells, %^#^	0.3 (0.1 – 0.4)
CD14^+^ monocytes, %^#^	0.3 (0.1 – 0.7)

Values are provided as median (25% - 75% interquartile range) if not otherwise stated; # as portion of CD45^+^ cells purified from the cerebrospinal fluid; expanded disability status scale (EDSS), immunoglobulin (Ig), cluster of differentiation (CD).

We included 86 patients into the clinical cohort with a median age of 34 (26 – 41) years, a disease duration of 4.5 (2.0 – 8.3) months, and mild clinical affection with a median EDSS of 1.0 (0 – 2.0) (**Table 2**). We excluded 31 eyes from further analysis due to a history of clinical ON and another 6 eyes due to suspected unilateral subclinical ON. Another 17 eyes were excluded due to poor OCT-A quality, resulting in 118 eyes of 86 patients that were included into the study (**Supplementary Figure 2**). Here, OCT-A measures at baseline were available in 82 of 86 patients and OCT values in all 86 patients. As defined in our study inclusion criteria, no patient suffered from an acute clinical relapse at study inclusion but the majority of patients (75/86, 87%) received a DMT. Thirty patients (35%) of the clinical cohort were also included into the CSF cohort, however, baseline examinations occurred at different time points. None of our patients from both cohorts have already been reported in other studies.

**Table 2:** Clinical characteristics of the clinical cohort.

Clinical cohort (n=86)	
Demographics	
Age, years	34 (26 – 41)
Sex female, No. (%)	50 (58)
Disease duration, months	5 (2 – 8)
EDSS	1.0 (0 – 2.0)
Diagnosis, No. (%)	
- Clinically isolated syndrome	5 (6)
- Relapsing remitting multiple sclerosis	81 (94)
Disease modifying therapy, No. (%)	
- no therapy	11 (13)
- category 1	57 (66)
- category 2	10 (12)
- category 3	8 (9)

Values are provided as median (25% - 75% interquartile range) if not otherwise stated; expanded disability status scale (EDSS).

We included a total of 40 age- and sex-matched healthy controls from the existing TUM OCT HC cohort. We excluded 9 eyes due to poor OCT-A quality and thus used 40 healthy individuals and 71 eyes for further analysis. A detailed flow diagram on person recruitment and data exclusion can be found within the **Supplementary Figure 3**.

As shown in **Table 3**, all three cohorts were balanced concerning age and sex. As expected, patients with MS from both cohorts revealed thinner GCIP values and a rarefication of superficial retinal vessels in eyes without a history of ON (**Table 3**), but comparable vessel densities within the DVC. Moreover, a worse HCVA and LCVA was detected in both MS cohorts as compared to HC. Both MS cohorts revealed similar clinical characteristics and retinal layer thicknesses as compared to other published cohorts of patients with early-stage relapsing remitting multiple sclerosis and clinically isolated syndrome (3, 4, 18, 19).

**Table 3:** OCT, OCT-A diagnostics and visual function in all study cohorts at study enrollment.

	CSF cohort (n=90 patients)	Clinical cohort (n=86 patients)	HC cohort (n=40 persons)	*p*-value
Age, years	35 (29 – 46)	34 (26 – 41)	34 (25 – 46)	0.34
Sex female, No. (%)	62 (69)	50 (58)	27 (68)	0.30
pRNFL, µm	99.8 ± 11.4	99.3 ± 13.5	103.3 ± 6.8	0.18
GCIP, µm	70.2 ± 5.9	69.7 ± 6.5	73.0 ± 4.7	0.02^a^
INL, µm	35.1 ± 2.3	34.7 ± 2.5	34.7 ± 2.5	0.42
SVC, % vessel density	25.4 ± 2.7	26.2 ± 2.8	27.6 ± 2.1	0.0001^b^
DVC, % vessel density	25.8 ± 2.7	26.3 ± 2.1	26.8 ± 1.8	0.17
FAZ, mm²	0.27 ± 0.11	0.24 ± 0.10	0.23 ± 0.10	0.09
HCVA	0.90 (0.74 – 1.00)	1.00 (0.90 – 1.13)	1.0 (0.90 – 1.43)	0.001^c^
LCVA	0.25 (0.16 – 0.32)	0.25 (0.20 – 0.33)	0.32 (0.23 – 0.45)	0.01^d^

Values are provided as mean ± standard deviation or median (25% - 75% interquartile range); one-way ANOVA or Kruskal-Wallis test depending on values are normally distributed or not; a: CSF vs. HC cohort p=0.03, Clinical vs. HC cohort p=0.02; b: CSF vs. HC cohort p<0.0001, Clinical vs. HC cohort p=0.02; c: CSF vs. Clinical cohort p=0.04, CSF vs. HC cohort 0.002; d: CSF vs. HC cohort p=0.007, CSF vs. HC cohort 0.04; cerebrospinal fluid (CSF), healthy control (HC), peripapillary retinal nerve fiber layer (pRNFL), common ganglion cell and inner plexiform layer (GCIP) and inner nuclear layer (INL) as measured by optical coherence tomography (OCT); superficial (SVC) and deep vascular complex (DVC) and foveal avascular zone (FAZ) as measured by OCT angiography.

### Association of the retinal vasculature and intrathecal immunity

**Figure 1:**
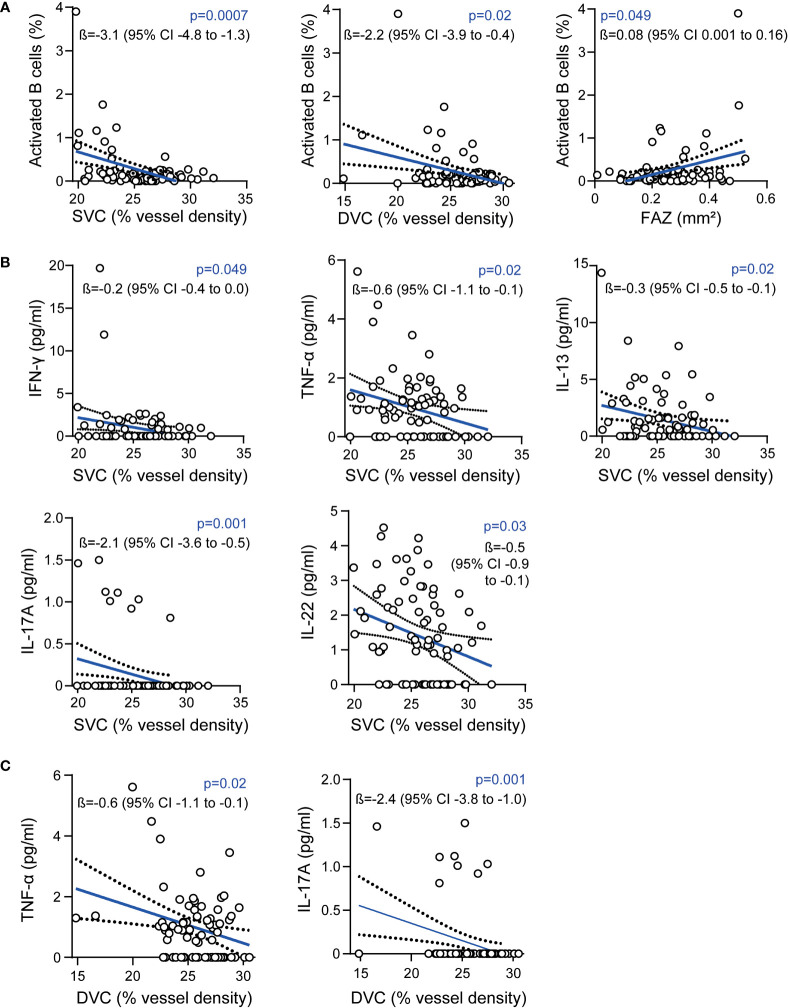
Association of the retinal vasculature and intrathecal immunity **(A)** Association of vessel densities of the superficial vascular complex (SVC), deep vascular complex (DVC), the foveal avascular zone (FAZ) and frequencies of intrathecal activated B cells; **(B)** Association of intrathecal cytokine levels and vessel densities of the SVC; **(C)** Association of intrathecal cytokine levels and vessel densities of the DVC; **(A–C)**: 87 patients, multiple linear regression models corrected for age and sex; ß estimates and 95% confidence (CI) intervals; interferon-γ (IFN-γ), tumor necrosis factor α (TNF-α), interleukin (IL).

In a first step, we investigated potential associations between OCT-A measures and the cellular intrathecal immune compartment in patients from the CSF cohort. As expected (3), GCIP thickness was negatively correlated with CSF frequencies of CD27^+^CD38^high^ activated B cells (ß=-3.0, 95% confidence interval [CI] -5.6 to -0.5, p=0.02, multiple linear regression model corrected for age and sex) and by trend with the total B cell frequency (ß=-0.5, 95% confidence interval [CI] -1.0 to 0, p=0.06). Moreover, we found the vessel densities in the SVC and DVC negatively correlated and the sizes of the FAZ positively correlated with the intrathecal frequency of activated B cells (**Figure 1A** ). Here, the association of vessel loss of both the SVC and DVC to a pronounced B cell mediated immunity in the CSF remained robust when additionally correcting for GCIP thicknesses (SVC: ß=-2.9, 95% CI -4.6 to -1.2, p=0.001; DVC: ß=-2.1, 95% CI -3.8 to -0.3, p=0.02; multiple linear regression models corrected for age, sex, disease duration, EDSS and GCIP thickness). Of note, the negative correlation of GCIP thicknesses and CSF frequencies of CD27^+^CD38^high^ activated B cells got lost when additionally correcting for SVC (ß=-0.9, 95% CI -3.5 to 5.3, p=0.69) and DVC vessel densities (ß=-1.1, 95% CI -5.6 to 3.4, p=0.62). We did not recognize any adjusted and significant association between SVC/DVC and frequencies of CD4^+^ T cells, CD8^+^ T cells, plasma cells, monocytes and both CD56^high^ and CD56^dim^ NK cells (data not shown).

Next, we addressed the humoral CSF immune compartment by analyzing different cytokines and chemokines. Here, the addressed cytokines were detectable in varying portions of patients. Of note, both BAFF and sCD40L were not detectable in 89 (99%) of 90 patients and were removed from the analysis. IL-17 was traceable in only 8 (9%), IL-21 in 11 (12%) and IL-2 in 12 (13%) of 90 patients whereas the remaining cytokines were traceable in 23% to 100% of patients (**Supplementary Table 2**). Here, vessel loss within the SVC was associated with higher CSF levels of the pro-inflammatory cytokines IFN-γ and TNF-α, as well as with IL-13 and IL-22 (**Figure 1B**). Furthermore, we found an association of lower vessel densities of the DVC and higher intrathecal levels of TNF-α (**Figure 1C**). In both the SVC (**Figure 1B**) and the DVC (**Figure 1C**), comparable associations were evident for SVC and DVC rarefication and higher IL-17 CSF levels, whereas this was driven by only 8 patients (see above). We did not recognize any adjusted significant association between SVC, DVC and FAZ measures and CSF levels of the remaining cytokines (data not shown).

When excluding all patients from the CSF cohort, that were also included into the clinical cohort, the majority of findings remained robust (**Supplementary Figure 4).** We did not detect any adjusted association of CSF Ig levels with any OCT or OCT-A results. Taken together, these data suggest that retinal vessel loss might be linked to a pro-inflammatory CSF phenotype during early-stage multiple sclerosis.

### Retinal vessel measurements as predictors for disability worsening

As a next step, we investigated potential associations of the retinal vasculature and the disease course in patients from the clinical cohort. The median follow-up period was 23 (14 – 20) months and the median time interval between two visits was 3 (2 – 4) 25 months. During that time, 9 patients (10%) from the clinical cohort suffered from sustained disability worsening, and 42 (49%) presented with ongoing MRI disease activity and 17 (20%) faced another clinical relapse.

Applying Cox proportional hazard regression models corrected for age, sex, disease duration, and immunotherapy during follow-up, a reduction in vessel densities of both the SVC and DVC was associated with a higher risk for future disability worsening (**Table 4**). As expected (3), a comparable association was evident for GCIP atrophy (**Table 4**). When additionally correcting for GCIP thickness, reduced SVC but not DVC vessel densities were linked to future disability worsening (HR 1.45, 95% CI 1.01 – 2.21, p=0.048). Interestingly, when additionally correcting for SVC vessel densities, GCIP atrophy lost its significance regarding future disability worsening (HR 1.2, 95% CI 1.0 – 1.5, p=0.07). We did not recognize any significant association of the SVC, DVC, and FAZ with MRI disease activity and occurrence of relapse (**Table 4**). When excluding all patients from the clinical cohort, that were also included into the CSF cohort, the association of SVC and DVC thinning of disability worsening remained robust (**Supplementary Table 3**).

**Table 4:** OCT-A measures associated with disability and disease activity.

Variable	Disability worsening		MRI disease activity		Relapse	
	HR (95% CI)	p	HR (95% CI)	p	HR (95% CI)	p
SVC^1^	1.56 (1.15–2.31)	0.01	0.93 (0.82-1.06)	0.27	0.91 (0.76-1.09)	0.34
DVC^1^	1.58 (1.05-2.50)	0.04	1.00 (0.83-1.19)	0.96	0.85 (0.65-1.10)	0.23
FAZ^1^	1.00 (1.00-1.00)	0.86	1.00 (1.00-1.00)	0.88	1.00 (1.00-1.00)	0.87
pRNFL^2^	1.04 (0.98-1.09)	0.18	1.02 (0.99-1.05)	0.20	1.03 (0.99-1.06)	0.11
GCIP^2^	1.24 (1.06-1.48)	0.01	1.01 (0.96-1.08)	0.65	1.07 (0.98-1.17)	0.13
INL^2^	1.04 (0.79-1.40)	0.74	1.12 (0.98-1.28)	0.10	1.12 (0.91-1.41)	0.27

Cox proportional hazard models for optical coherence tomography angiography (OCT-A) predictors on disability worsening, MRI disease activity and occurrence of relapse adjusted for age, sex, disease duration and disease modifying therapy. (1) Decrease of 1% point in vessel density; (2) decrease of 1 µm in layer thickness. Hazard ratio (HR) with 95% confidence intervals (CI); superficial vascular complex (SVC), deep vascular complex (DVC) and foveal avascular zone (FAZ) as measured by OCT-A; peripapillary retinal nerve fiber layer (pRNFL), common ganglion cell layer (GCIP) and inner nuclear layer (INL) as measured by optical coherence tomography.

**Figure 2:**
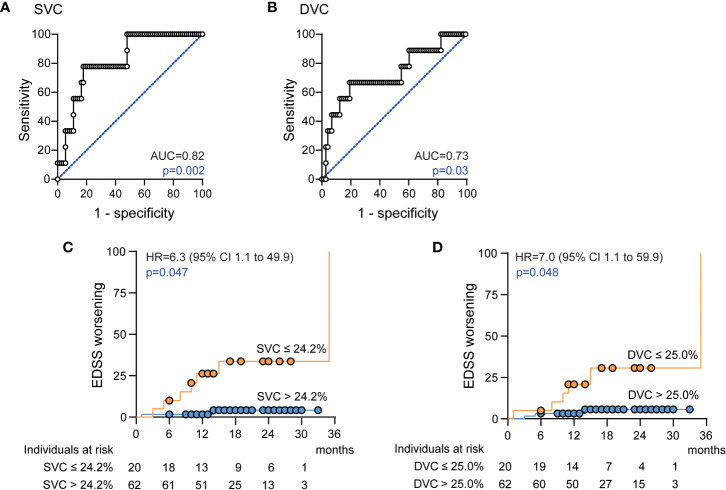
Association of the retinal vasculature and disability worsening **(A)** Receiver operating characteristics (ROC), ROC curve and area under the curve (AUC) measures for superficial vascular complex (SVC) vessel densities; **(B)** ROC curve and AUC measures for deep vascular complex (DVC) vessel densities; **(C)** Cumulative fraction of patients with confirmed disability worsening stratified by SVC vessel densities ≤ 24.2% and > 24.2% at baseline; **(D)** Cumulative fraction of patients with confirmed disability worsening stratified by DVC vessel densities ≤ 25.0% and > 25.0% at baseline; **(C, D)**: Kaplan-Meier plots with Cox proportional hazards regression adjusted for age, sex, disease duration, disease modifying therapy and thickness of the common ganglion cell and inner plexiform layer. Hazard ratio (HR), confidence interval (CI).

Having found SVC and DVC as possible biomarkers for risk stratification of future disability worsening, we applied additional ROC analyses to identify possible discriminatory values. Here, a SVC vessel density of ≤ 24.2% revealed a sensitivity of 77.8% and a specificity of 79.5% (AUC 0.82, Harrel’s C 0.86) and DVC vessel measures of ≤ 25.0% a sensitivity of 66.7% and a specificity of 80.8% (AUC 0.73, Harrel’s C 0.84) to identify patients with future disability worsening (**Figures 2A, B**). Here, SVC measures ≤ 24.2% were evident in only 1 (2.5%) and DVC measures ≤ 25.0% in only 6 (15%) of 40 HC. When applying these discriminatory values into the clinical cohort, a total of 20 of 82 (24%) patients revealed SVC densities ≤ 24.2% as compared to 62 patients (76%) with higher measures. Demographics and exposure to immunomodulatory therapy were comparable between both groups whereas GCIP thickness was significantly lower in patients with low SVC vessel densities (**Table 5**). Patients with SVC vessel densities ≤ 24.2% revealed a 6.3-fold increased risk for disability worsening during a follow-up period of up to 33 months when correcting for age, sex, disease duration, immunotherapy and GCIP thickness (**Figure 2C**). In line with this, 20 of 82 patients (24%) had DVC measures ≤ 25.0% and revealed comparable demographics and DMT exposure as patients with higher DVC vessel densities (data not shown) and similar GCIP thicknesses (DVC ≤ 25.0%: GCIP 69.1 ± 7.0; DVC > 25%: GCIP 70.0 ± 6.2; p=0.58). Patients with DVC measures of ≤ 25.0% revealed a 7.0-fold risk for disability worsening as compared to patients with higher measures (**Figure 2D**). Taken together, our data suggest that retinal vessel loss within both the SVC and DVC might be linked to future disability worsening in patients with early multiple sclerosis.

**Table 5:** Demographics and OCT parameters in patients with SVC vessel densities of ≤ 24.2% and > 24.2%.

	SVC ≤ 24.2%(n=20 patients)	SVC > 24.2%(n=62 patients)	*p*-value
**Demographics**			
Age, years	34 (26 – 46)	34 (26 – 40)	0.69
Sex female, No. (%)	15 (75)	35 (53)	0.08
Disease duration, months	3 (1 – 8)	6 (2 – 9)	0.12
EDSS	0.5 (0 – 2.0)	1.0 (0 – 2.0)	0.33
Diagnosis, No. (%)			0.57
- Clinically isolated syndrome	0 (0)	4 (6)	
- Relapsing remitting multiple sclerosis	20 (100)	94 (92)	
Disease modifying therapy, No. (%)			0.25
- no therapy	2 (10)	8 (13)	
- category 1	12 (60)	43 (69)	
- category 2	2 (10)	7 (11)	
- category 3	4 (20)	4 (6)	
**OCT parameters**			
pRNFL, µm	96.8 ± 11.2	100.1 ± 13.3	0.31
GCIP, µm	66.5 ± 5.6	70.9 ± 6.2	0.006
INL, µm	34.7 ± 2.3	34.7 ± 2.4	0.98

## Discussion

Our study aims to integrate alterations of the retinal vasculature into the pathophysiological concept of multiple sclerosis. Our data indicate a possible relation of retinal vessel loss, a pro-inflammatory intrathecal immune phenotype, and poor disease prognosis in patients with early multiple sclerosis. Thus, OCT-A-based evaluation of the retinal vasculature may add valuable aspects to the therapeutical management of patients with early multiple sclerosis.

During the last years, the OCT-A technique has been applied by us (6, 8, 15) and others (7, 20, 21) to study alterations of retinal vasculature in patients with multiple sclerosis. Here, superficial vessel loss has been shown to be a consistent finding in patients with relapsing remitting multiple sclerosis and a history of ON (7, 8, 20, 21) and we could recently show, that rarefication of the SVC but not the DVC occurs within the first three months after acute ON and evolves simultaneously to GCIP atrophy (6). There is growing evidence, however, that alterations of the retinal vasculature might also occur in eyes without a history of ON during the disease course of multiple sclerosis which was confirmed by the present study. SVC rarefication in eyes without a history of clinical ON has been reported by several groups during the last years (22, 23). Lanzillo et al. (24) described thinning of the retinal parafoveal vasculature (without differentiating into different vascular plexus) which was linked to higher EDSS scores. In line with this, a decline of the SVC but not the DVC occurred in eyes of patients with multiple sclerosis without a history of ON and was associated with higher disability scores and poor visual acuity in a study from Murphy et al. (7). These findings suggest that ON-unrelated rarefication of the retinal vasculature might be associated with pronounced disability during multiple sclerosis.

In our study, an intrathecal pro-inflammatory phenotype was linked to an atrophy of retinal ganglion cells and decreased retinal perfusion measurements within the SVC and DVC. These findings are in line with the literature. We could show in the past that ganglion cell loss during multiple sclerosis might be linked to a pronounced humoral immune response within the CSF (3) and a recent publication suggested that CSF pleocytosis is associated with retinal axonal loss (25). Moreover, there is first evidence that retinal OCT might also allow to monitor microglia function and immunity during MS. Retinal hyperreflective foci, which are mainly found within the inner nuclear layer and are considered as clustered retinal microglia, have been linked to elevated levels of several pro-inflammatory cytokines within the CSF (26). Studies addressing both OCT-A and CSF measurements are lacking and our study is the first one that links retinal vessel loss to a pro-inflammatory intrathecal immune phenotype. Taken together, these data suggest that the retina might be capable to mirror the intrathecal immunity during multiple sclerosis.

As another feature, our present study provides evidence that OCT-A-based analysis of the retinal vasculature might allow risk stratification of patients with early relapsing remitting multiple sclerosis. There is a huge amount of literature by us (3, 4, 27) and others (18, 28–31), suggesting that both retinal axonal and ganglion cell loss as measured by conventional OCT is associated with a worse disease prognosis and disability worsening in multiple sclerosis. To the best of our knowledge, this the first study to apply OCT-A for prognostication of disease activity in patients with multiple sclerosis. As already discussed above, decreased retinal vessel densities have been linked to worse visual acuity and pronounced disability in cross-sectional studies (7). In line with this, a longitudinal parafoveal vessel loss has been described to be linked to increasing EDSS scores (32) whilst increased retinal vessel densities might be associated with a stable disease course and improved visual function (33). Thus, retinal OCT-A might be a useful tool to predict the future disease course in patients with early relapsing remitting multiple sclerosis.

The underlying mechanisms leading to alterations of the retinal vasculature in patients with multiple sclerosis remain unclear. We could recently show that retinal vessel loss within the SVC following acute ON occurs simultaneously to ganglion cell atrophy (6). Since the SVC supplies the RNFL and GCIP, SVC thinning could be a secondary effect of GCIP atrophy due to a reduced metabolic activity and a lower oxygen demand. This theory could be supported by the fact that GCIP atrophy during multiple sclerosis has also shown to be linked to a pro-inflammatory CSF phenotype and future disability worsening (3). In our study, however, the majority of our findings remained statistically robust when additionally correcting for GCIP thicknesses. In contrast, the associations of GCIP atrophy with a pro-inflammatory CSF-phenotype and future disability worsening got lost when additionally correcting for SVC vessel densities. This suggests, that GCIP loss does not completely explain SVC thinning and its association with the disease course and intrathecal immunity in patients with relapsing remitting multiple sclerosis. Our data also might raise the hypothesis, that GCIP loss might occur secondary to retinal vessel rarefication, which needs to be tested in future studies.

On the contrary, ON-independent SVC loss could be the consequence of inflammatory processes within the retina. The occurrence of retinal periphlebitis in multiple sclerosis has been described more than 30 years ago and might be linked to disruption of both the blood-brain- and the blood-retinal-barrier (34). Retinal astrocytic Müller cells, together with retinal microglia, pericytes, and endothelial cells are essential for the composition and integrity of the blood-retinal-barrier which furthermore modulates the retinal blood supply (35). Although retinal periphlebitis with infiltration of immune cells into the retina is a very rare finding, autopsy studies have described hypertrophy and decline of Müller cells with activation of retinal microglia in proximity to vessels as a common finding in the majority of multiple sclerosis patients (36). Loss of tight junctions within the retinal vasculature clearly suggests an alteration and disruption of the blood-retinal-barrier unrelated to any evident retinal infiltration of immune cells (36). We could recently show that retinal vessel loss occurs subclinically in patients with neuromyelitis optica spectrum disorders and antibodies against aquaporin-4. Here, rarefication of the SVC was linked to higher serum levels of the glial fibrillary acidic protein (GFAP) indicating a pronounced damage of astrocytes (15). Using the murine MS model of experimental autoimmune encephalomyelitis, we could recently show that aquaporin-4 expressed by retinal Müller cells is essential for the integrity of the gliovascular unit and that genetic ablation of aquaporin-4 results in a disturbed retinal perfusion (10). Therefore, changes of the retinal vasculature could be the consequence of inflammatory alterations of retinal glia cells. By establishing a possible association of retinal vessel loss and a pro-inflammatory intrathecal immune phenotype, our study provides further evidence for this theory.

Our study has several limitations. Firstly, the longitudinal follow-up period of our study is short and only a small portion of patients faced disability worsening. These circumstances might lead to false positive results and our findings thus need to be discussed with caution. OCT-A, however, is a novel technique and longer follow-up durations are thereby limited. Secondly, the results of our CSF analysis are limited by the fact that some cytokines – especially IL-17 – were only traceable in a small portion of patients. Thirdly, OCT-A is technically challenging. Especially for eyes with visual impairment, OCT-A becomes extremely susceptible to imaging artifacts that affect vessel density measurements (37). All examinations were conducted by experienced technicians, and we used a rigorous approach for OCT-A quality control to ensure reliable OCT-A results. Widely accepted OCT-A quality criteria, however, are missing to date. Fourthly, OCT-A measurements are device-specific (38). We cannot exclude a device-specific effect on our results and conclusions, and studies using and comparing different OCT-A machines are needed. Fifthly, there are methodological issues that restrict the interpretability of our findings. OCT-A provides information about retinal perfusion patterns, but not on vessel morphology and vessel integrity. An automatic and robust differentiation of retinal vessel structures into veins and arteries is not possible and the currently used technique does not allow to distinguish whether a decrease in retinal vessel density reflects true vessel loss, constriction, or shrinkage of vessels. Here, advances in both hardware and software solutions are urgently needed. Sixthly, the physiological influence of age and sex and other environmental factors on OCT-A measures are focus of current research and thus not entirely clear. It is probable that age especially might influence retinal vessel densities and that our finding might thus not transferable of younger or older patient cohorts. However, to address this issue, we corrected all our statistical models for the covariate age to reduce an influence of purely age-related changes on our findings.

Taken together, our study suggests an association of the retinal vasculature, intrathecal immunity, and disease course in patients with multiple sclerosis. If confirmed by others and validated within bigger trials with longer clinical follow-up durations, evaluation of the retinal vasculature could allow for estimating disease activity and prognosis.

## Data availability statement

Data are available upon reasonable request from the corresponding author. We will share raw imaging OCT-A data in an anonymized way upon request by any qualified investigator. The data are not publicly available due to privacy or ethical restrictions.

## Ethics statement

The studies involving human participants were reviewed and approved by Ethics committee of the Technical University of Munich, School of Medicine. The patients/participants provided their written informed consent to participate in this study.

## Author contributions

CN collected and interpreted data, had a major role during data analysis and revised the manuscript for intellectual content. MH contributed to acquisition and interpretation of data and revised the manuscript for intellectual content. LA contributed to acquisition and interpretation of data and revised the manuscript for intellectual content. RW contributed to acquisition and interpretation of data. AA contributed to acquisition and interpretation of data. CM contributed to acquisition and interpretation of data and provided resources. CG contributed to acquisition and interpretation of data and revised the manuscript for intellectual content. AB contributed to acquisition and interpretation of data and revised the manuscript for intellectual content. BH contributed to the conceptualization of the study, interpretation of data, revised the manuscript for intellectual content and provided resources. TK contributed to the conceptualization of the study, interpretation of data and revised the manuscript for intellectual content. BK conceptualized and designed the study, contributed to data interpretation and data analysis, and drafted the manuscript. All authors contributed to the article and approved the submitted version.

## Funding

CN received a research scholarship from the Gemeinnützige Hertie Foundation (medMS program). RW received an intramural research grant from the Technical University of Munich, School of Medicine. AA received intramural funding from the Technical University of and was supported by the Deutsche Forschungsgemeinschaft (DFG, German Research Foundation) under Germany’s Excellence Strategy within the framework of the Munich Cluster for Systems Neurology (EXC 2145 SyNergy – ID 390857198). CG was supported by the Hertie Network of Clinical Neuroscience, the Hans und Klementia Langmatz Stiftung, and the German Federal Ministry of Education and Research (BMBF, 161L0216). BH received funding for the study from the MultipleMS EU consortium, the Clinspect-M consortium funded by the Bundesministerium für Bildung und Forschung and the Deutsche Forschungsgemeinschaft (DFG, German Research Foundation) under Germany’s Excellence Strategy within the framework of the Munich Cluster for Systems Neurology (EXC 2145 SyNergy – ID 390857198). BH is associated with DIFUTURE (Data Integration for Future Medicine, BMBF 01ZZ1804[A-I]). TK was supported by the Deutsche Forschungsgemeinschaft (SFB1054-B06 (ID 210592381), TRR128-A07 (ID 213904703), TRR128-A12 (ID 213904703), TRR128-Z02 (ID 213904703), TRR274-A01 (ID 408885537), and EXC 2145 (SyNergy, ID 390857198), the ERC (CoG 647215), and by the Hertie Network of Clinical Neuroscience. BK is funded by the Else Kröner-Fresenius-Stiftung (Else Kröner-Fresenius Exzellenzstipendium 2019_EKES.09), the Gemeinnützige Hertie Foundation (medMS program) and received a research award from Novartis (Oppenheim award 2020). The funder was not involved in the study design, collection, analysis, interpretation of data, the writing of this article or the decision to submit it for publication.

## Acknowledgments

We thank Mira Radic and Andrea Hennemann for expert assistance during OCT-A acquisition and analysis and Iaroslav Zinevych, Regina Kohler, Petra Kirchpfennig and Anna-Maria Lourie for assistance during CSF analysis. Biosamples were taken from Biobank resources at the Department of Neurology at Technical University of Munich, Germany, which is part of the Joint Biobank Munich in the framework of the German Biobank node.

## Conflict of interest

LA received travel and research support by Novartis. CM serves as medical advisor at Heidelberg Engineering. AB has received consulting and/or speaker fees from Alexion, Bayer Healthcare, Biogen, Celgene, Novartis, Roche and Sandoz/Hexal. His institution has received compensation for clinical trials from Alexion, Biogen, Merck, Novartis, Roche, and Sanofi Genzyme - all outside the reported work. BH served on scientific advisory boards for Novartis; he has served as DMSC member for AllergyCare, Sandoz, Polpharma and TG therapeutics; he or his institution have received speaker honoraria from Desitin; his institution received research grants from Regeneron for multiple sclerosis research. He holds part of two patents; one for the detection of antibodies against KIR4.1 in a subpopulation of patients with multiple sclerosis and one for genetic determinants of neutralizing antibodies to interferon. BK received travel support and a research grant from Novartis and speaker honoraria from TEVA.

The remaining authors declare that the research was conducted in the absence of any commercial or financial relationships that could be construed as a potential conflict of interest.

## Publisher’s note

All claims expressed in this article are solely those of the authors and do not necessarily represent those of their affiliated organizations, or those of the publisher, the editors and the reviewers. Any product that may be evaluated in this article, or claim that may be made by its manufacturer, is not guaranteed or endorsed by the publisher.
