# Supplementary material for: Association of the retinal vasculature, intrathecal immunity, and disability in multiple sclerosis
Source: Front Immunol. 2022 Nov 11;13:997043. doi: 10.3389/fimmu.2022.997043 (PMC9695398; doi:10.3389/fimmu.2022.997043)
Supplement: Supplementary file 1 [file DataSheet_1.pdf]

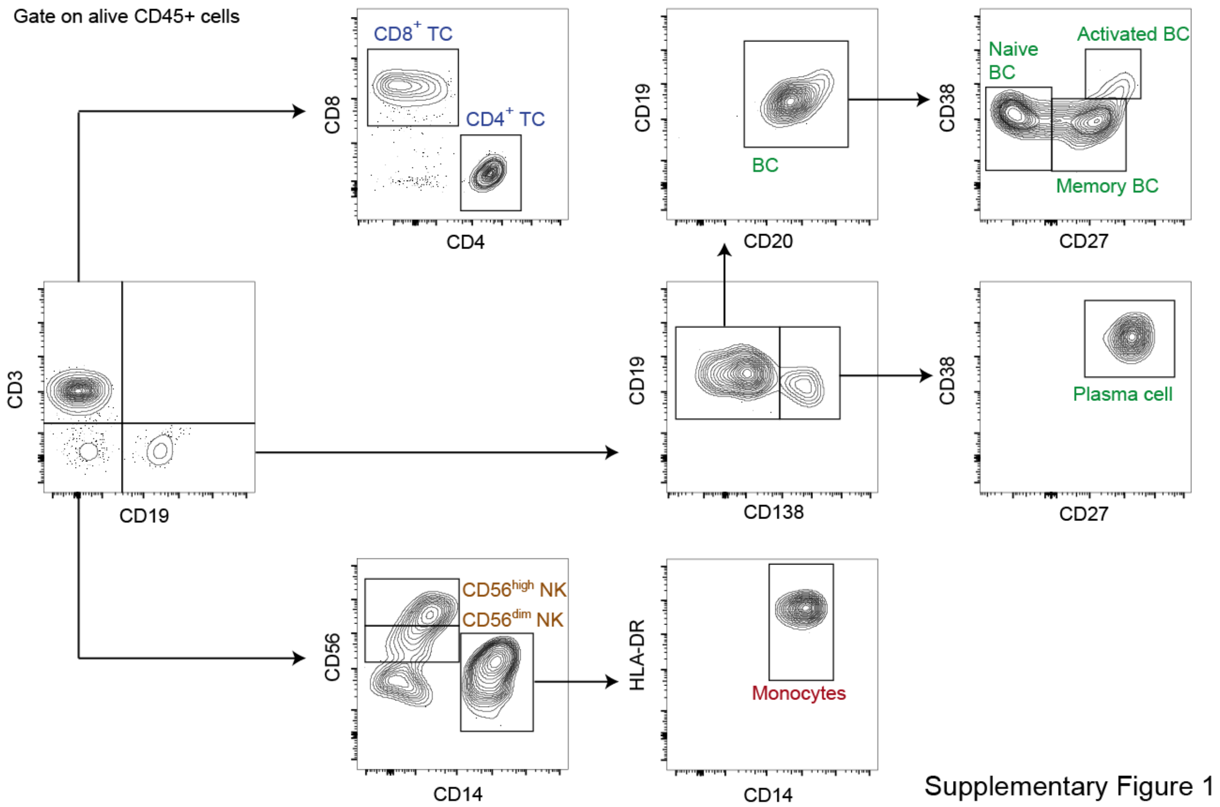

Supplementary Figure 1: Gating strategy for analysis of the intrathecal cellular immune compartment

Gate on $CD45^+$ alive cells purified from the cerebrospinal fluid. Frequencies of CD8+ T cells ($CD8^+$ TC), CD4+ T helper cells ($CD4^+$ TC), naïve B cells (naïve BC), memory B cells (Memory BC), activated B cells (activated BC), plasma cells, $CD56^{high}$ and $CD56^{dim}$ natural killer cells (NK) and monocytes were analyzed; cluster of differentiation (CD).

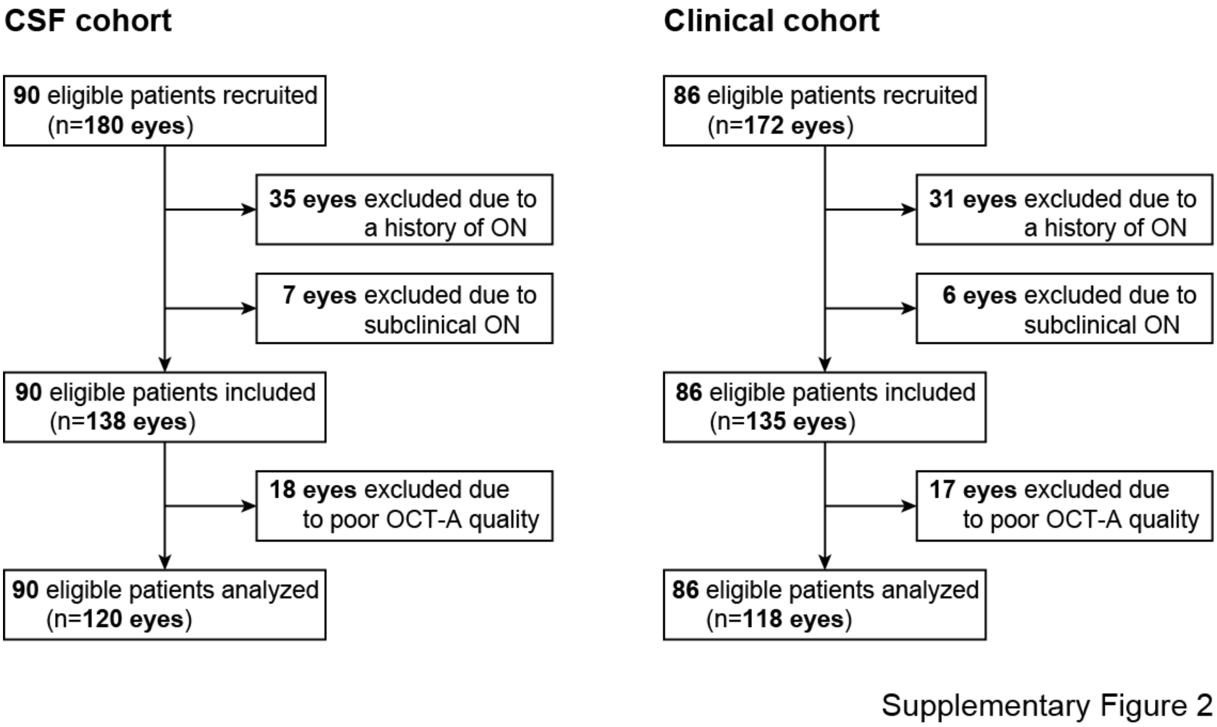

Supplementary Figure 2: Flow diagram of patient recruitment and data exclusion in both multiple sclerosis cohorts

Cerebrospinal fluid (CSF) cohort and clinical cohort of patients with relapsing remitting multiple sclerosis and clinically isolated syndrome.

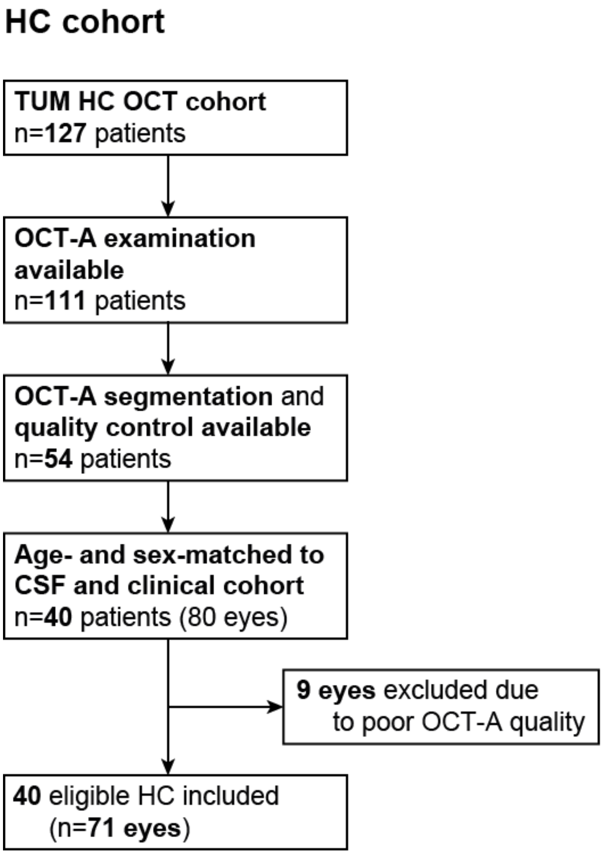

Supplementary Figure 3

Supplementary Figure 3: Flow diagram of healthy control recruitment and data exclusion

Technical University of Munich (TUM) healthy control (HC) optical coherence tomography (OCT) cohort; OCT angiography (OCT-A)

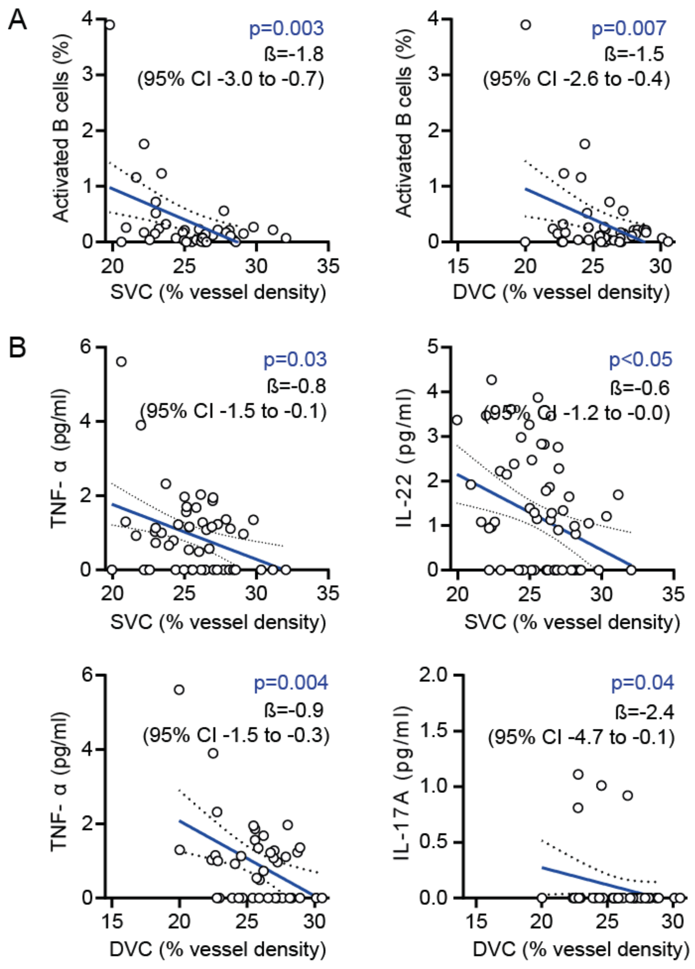

Supplementary Figure 4

Supplementary Figure 4: Association of the retinal vasculature and intrathecal immunity when excluding patients from the clinical cohort

(A) Association of vessel densities of the superficial vascular complex (SVC) and deep vascular complex (DVC) and frequencies of intrathecal activated B cells; (B) Association of intrathecal cytokine levels and vessel densities of the SVC and DVC; multiple linear regression models corrected for age and sex; ß estimates and 95% confidence (CI) intervals; interferon-γ (IFN-γ), tumor necrosis factor α (TNF-α), interleukin (IL).

**Site of relapse (n=77)**	
Optic neuritis, No. (%)	32 (42)
Myelitis, No. (%)	23 (30)
Other, No. (%)	22 (28)
**Neurological systems affected (n=77)**	
Pyramidal system	12 (16)
Sensory system	43 (56)
Cerebellum	3 (4)
Brainstem	9 (12)
Bladder / bowel	1 (1)
Cerebral	2 (3)

Supplementary Table 1: Relapse type and affected neurological systems in patients suffering from an acute relapse from the CSF cohort (n=77)

Neurological systems affected by relapse do not sum up to 100% since some relapses affected more than one neurological system.

	**CSF level (pg/ml)** median (25% - 75% interquartile range)#	**Samples with detectable target** No. (%)	**Minimal detectable concentration** (pg/ml)+
IL-2	0.9 (0.5 - 2.0)	12 (13%)	0.44
IL-4	1.1 (1.0 - 1.2)	26 (28%)	1.12
IL-5	4.1 (3.2 - 4.7)	27 (30%)	2.51
IL-6	1.6 (1.0 - 3.0)	66 (73%)	0.74
IL-9	2.3 (1.8 - 3.4)	74 (82%)	1.64
IL-10	0.9 (0.6 - 1.4)	21 (23%)	0.77
IL-13	1.6 (0.8 - 3.4)	45 (50%)	0.39
IL-17A	1.1 (0.9 - 1.4)	8 (8%)	0.98
IL-17F	0.7 (0.6 - 0.8)	23 (25%)	0.31
IL-21	1.4 (1.1 - 4.1)	11 (12%)	1.51
IL-22	2.1 (1.2 - 2.8)	52 (57%)	1.45
IFN-γ	1.8 (1.0 - 2.4)	27 (30%)	0.68
TNF-α	1.2 (1.0 - 1.7)	51 (56%)	0.47
APRIL	36.5 (23.8 - 54.8)	90 (100%)	0.87
BAFF	36.7	1 (1%)	0.96
sCD40L	0.6	1 (1%)	0.26

Supplementary Table 2: Results from flow cytometry-based multiplex assays of the CSF

only CSF samples considered with levels > minimal detectable concentration; + as suggested by the manufacturer

Variable	Disability worsening	
	HR (95% CI)	p
**SVC**[1]	2.04 (1.33–4.16)	**0.008**
**DVC**[1]	2.18 (1.26-4.81)	**0.01**
**FAZ**[1]	1.00 (1.00-1.00)	0.76
**pRNFL**[2]	1.03 (0.95-1.13)	0.48
**GCIP**[2]	1.08 (0.91-1.32)	0.28
**INL**[2]	1.03 (0.67-1.51)	0.89

Supplementary Table 3: OCT-A measures associated with disability when excluding patients from the CSF cohort

Cox proportional hazard models for optical coherence tomography angiography (OCT-A) predictors on disability worsening adjusted for age, sex, disease duration and disease modifying therapy. (1) Decrease of 1% point in vessel density; (2) decrease of 1 µm in layer thickness. Hazard ratio (HR) with 95% confidence intervals (CI); superficial vascular complex (SVC), deep vascular complex (DVC) and foveal avascular zone (FAZ) as measured by OCT-A; peripapillary retinal nerve fiber layer (pRNFL), common ganglion cell layer (GCIP) and inner nuclear layer (INL) as measured by optical coherence tomography.
